# Current Susceptibility Surveillance and Distribution of Antimicrobial Resistance in *N. gonorrheae* within WHO Regions

**DOI:** 10.3390/pathogens11111230

**Published:** 2022-10-25

**Authors:** Marina Radovanovic, Dusan Kekic, Milos Jovicevic, Jovana Kabic, Ina Gajic, Natasa Opavski, Lazar Ranin

**Affiliations:** 1 Department of Biochemistry, Institute for Biological Research “Siniša Stanković”, University of Belgrade, Belgrade 11000, Serbia; 2 Institute of Microbiology and Immunology, Faculty of Medicine, University of Belgrade, Belgrade 11000, Serbia

**Keywords:** *Neisseria gonorrhoeae*, antimicrobial resistance, resistance genes, surveillance, WHO regions

## Abstract

*Neisseria gonorrhoeae* (*N. gonorrhoeae*) is the etiological agent of the second most common sexually transmitted disease in the world, gonorrhoea. Currently recommended and last available first-line therapy is extended-spectrum cephalosporins most often combined with azitromycin. However, misuse of antibiotics and the abilities of *N. gonorrhoeae* to acquire new genetic and plasmid-borne resistance determinants has gradually led to the situation where this bacterium has become resistant to all major classes of antibiotics. Together with a generally slow update of treatment guidelines globally, as well as with the high capacity of gonococci to develop and retain AMR, this may lead to the global worsening of gonococcal AMR. Since effective vaccines are unavailable, the management of gonorrhoea relies mostly on prevention and accurate diagnosis, together with antimicrobial treatment. The study overviews the latest results of mostly WHO-initiated studies, primarily focusing on the data regarding the molecular basis of the resistance to the current and novel most promising antibacterial agents, which could serve to establish or reinforce the continual, quality-assured and comparable AMR surveillance, including systematic monitoring and treatment with the use of molecular AMR prediction methods.

## 1. Introduction

*Neisseria gonorrhoeae* (also known as the gonococci) belongs to the genus *Neisseria*, consisting of another pathogenic species, *Neisseria meningitidis* (also known as the meningococci), and at least eight non-pathogenic commensal species, which represents a substantial proportion of the human nasal and oropharyngeal microbiota. *N. gonorrhoeae* and *N. meningitidis* primarily occupy two distinct respective niches: the genital and nasopharyngeal mucosa. *N. gonorrhoeae* is the etiological agent of the second most prevalent sexually transmitted infection (STI), gonorrhoea, while *N. meningitidis* is one of the leading causes of bacterial meningitis [1]. According to the World Health Organization (WHO), it is estimated that 78–87 million new cases of gonorrhoea are diagnosed each year worldwide [2]. Moreover, a worrying overall rise in gonorrhoea prevalence of up to 45% from 2016, with a vast proportion being men who have sex with men (MSM) [3]. Furthermore, *N. gonorrhoeae* may also be detected in rectal and oropharyngeal mucosa [4]. In men, gonorrhoea most commonly manifests as urethritis and epididymo-orchitis, while women typically develop asymptomatic cervicitis. Nevertheless, if left untreated, female infections may lead to pelvic inflammatory disease, ectopic pregnancies, and infertility. Moreover, complicated gonococcal infections stimulate human immunodeficiency virus acquisition and transmission by increasing its loads in the genital tract [5].

Since effective vaccines are not yet available, the management of gonorrhoea besides prevention relies on accurate diagnosis and antimicrobial treatment. Over the last century, *N. gonorrhoeae* has gradually acquired resistance to almost all major classes of antibiotics, including penicillins, macrolides, tetracyclines, and fluoroquinolones [4]. Thus, gonorrhoea treatment with the above-mentioned antibiotics was abandoned wherever possible, the currently recommended and last available first-line monotherapy represents extended-spectrum cephalosporins (ESC)—cefixime and ceftriaxone [1,4]. However, most data collected worldwide during the last decade indicate that *N. gonorrhoeae* is rapidly developing resistance to these antibiotics as well [6]. Namely, sporadic failures to cure pharyngeal gonorrhoea with ceftriaxone monotherapy have been confirmed in several countries [7,8]. Consistently, the international spread of ceftriaxone-resistant gonococci was confirmed in 2015–2019. Even before this, recommendations of dual therapy ceftriaxone and azithromycin, have been introduced in the EU and the US, but are now recommended by WHO guidelines and exploited wherever thorough, quality-assured local AMR surveillance data are missing [2]. The typical current treatment guidelines include a single oral or intramuscular dose of an ESC (400 mg per os of cefixime or 250–500 mg intramuscular ceftriaxone) in combination with a single oral dose of 1–2 g azithromycin [9,10,11]. The first strain with resistance to ceftriaxone combined with high-level azithromycin resistance was isolated in England and Australia in 2018 [12,13,14]. Fortunately, the cases of resistant gonorrhoea to ceftriaxone monotherapy or dual therapy with azithromycin remain rare, despite the international spread of the ceftriaxone-resistant FC428 clone, and a slight rise in the prevalence of strains with a decreased susceptibility or resistance to ceftriaxone and azithromycin [15]. Accordingly, dual-antimicrobial regimens might not be an effective long-term solution and are still not affordable in many resource-poor settings. Namely, in these settings, the ESCs are frequently unavailable, since they are relatively expensive even when used as monotherapeutics [6]. Thus, in order to evade the possible emergence of untreatable gonorrhoea worldwide, novel, commonly accessible, cost-effective and nontoxic therapeutics are direly needed. Two potential new antimicrobials have emerged as most promising: a DNA topoisomerase IV and DNA gyrase inhibitor, gepotidacin and zoliflodacin, which targets bacterial type II topoisomerases by a distinct mechanism from quinolones such as ciprofloxacin [15,16,17]. Both of these antimicrobial therapeutics are currently in phase 3 of randomised controlled clinical trials for the treatment of uncomplicated gonorrhoea [18].

The antimicrobial resistance capacity of gonococci has largely been stimulated by unrestricted availability and consequent over-use of antimicrobials worldwide. On the other hand, the emergence of the molecular resistance determinants in new gonococcal strains is also propelled by several species-specific characteristics. Most important is its high competence for transformation, driven both by the ability to take up gonococcal and other bacterial DNAs and plasmids from the environment, as well as to perform highly efficient recombination with the homologous gonococcal sequences [19,20,21]. Transformation may produce mosaic genes of *N. gonorrhoeae* that represent antimicrobial resistance determinants, wherein two similar genes combine to form a mosaic gene of the resistant variant. Additionally, mutations may also arise within a single gene, conferring antibiotic resistance to its product [22]. The presence of human commensal *Neisseria* spp. and the potential presence of genetic determinants of resistance in the corresponding homologous sequences may be of particular importance, especially from the point of view of *N. gonorrhoeae* fitness advances [1]. Namely, most confirmed failures of gonorrhoea treatment were described for pharyngeal infections, where multiple microbes have limited simultaneous access, infections are most frequently asymptomatic and new genetic AMR determinants may be acquired from coexisting, non-pathogenic *Neisseria* species [6].

## 2. Brief Description of the Most Important Mechanisms of Resistance in Gonococci

Principal molecular determinants of *N. gonorrhoeae* AMR include protectively altered antibiotic targets, multidrug efflux pumps and antibiotic deactivators [1]. One set of genomic alterations conferring antimicrobial resistance occurs in the target sites of genes. These alterations reduce the affinities and increase the corresponding resistance levels. Such are mutations in the penicillin-binding protein 2 (PBP2)-encoding gene *penA* which retain sufficient transpeptidase activity but reduce acylation by penicillin and cephalosporins, providing reduced susceptibility to these antibiotics [6]. Penicillin resistance is most commonly the result of aspartate insertions at specific positions while reduced susceptibility or resistance to cephalosporins is generally associated with alanine polymorphisms or, more importantly, mosaic *penA* alleles with multiple simultaneous polymorphisms. Strains displaying high-level ceftriaxone resistance also commonly contain mosaic *penA* alleles which allow them to spread internationally [6]. For instance, the globally transmitting FC428 clone which may have originated from commensal *Neisseria cinerea*, as well as the WHO Q strain with ceftriaxone resistance plus high-level azithromycin resistance, harbour mosaic *penA*-60.001 [23,24]. Recent biochemical and structural analyses of PBP2 variants from several *N. gonorrhoeae* strains with decreased susceptibility to ceftriaxone, indicate that mutations lower the ceftriaxone PBP2 affinity by restricting its conformational changes and flexibility that normally accompany acylation in such ways that may trap ceftriaxone in a non-canonical configuration or hinder its contact with the PBP active site [25]. As far as resistance-conferring mutations in the genes encoding for targets of other antimicrobials are concerned, the ones important for tetracycline resistance include polymorphisms in the genes encoding for the parts of the ribosomal 30S subunit one of which may be the *rpsJ* gene for the ribosomal protein S10. Spectinomycin also targets the 30S ribosome subunit, but its resistance results from C1192U mutation in the 16S rRNA or the mutations in ribosomal protein S5, encoded by *rpsE*, one of which is the frequent combination of valine deletion at position 25 and K26E polymorphism [6]. Similarly, since azithromycin targets the 50S ribosomal subunit by blocking the 23S rRNA component, various levels of resistance against azithromycin result from mutations and specific polymorphisms in the genes encoding for target loop V of 23S rRNA [6]. Finally, ciprofloxacin targets the DNA gyrase, consisting of GyrA and GyrB subunits, and the topoisomerase IV which consists of the ParC and ParE subunits. Thus, a great variety of specific mutations in the corresponding genes influence the susceptibility of *N. gonorrhoea* to this antibiotic, with the exact resistance levels being determined by the specific combination of these mutations [6].

A reduced influx of penicillin, cephalosporins, and tetracycline through changes in the major outer membrane porin PorB (encoded by *porB* gene), more specifically, mutations in the PorB constriction zone at positions G120 and A121 into bulkier charged residues, decrease susceptibility to these antibiotics [6]. One of the most important gonococcal antimicrobial resistance determinants that provide multidrug resistance to all hydrophobic or amphipathic drugs including penicillins, cephalosporins, azithromycin, tetracycline and ciprofloxacin is the multidrug efflux pump MtrC–MtrD-MtrE (encoded by *mtr*CDE) and its repressor MTR (encoded by *mtrR*) [6]. Genomic mutations that derepress its function, which cannot manifest independently as resistance determinants, but require concomitant mutations in the mtrR gene, include the deletions in the promoters of *mtrR* and *mtr*CDE genes, as well as specific polymorphisms of *mtrR* and mosaic *mtr*CDE alleles acquired from *N. meningitidis* and commensal *Neisseria* [26,27].

There are also non-genomic molecular determinants of resistance to antibiotics: the plasmid-borne genes for the antibiotic-inactivating enzyme, *bla*TEM (encoding for β-lactamase), and the transposon-borne class M tetracycline genes (*tetM*) which provide high-level resistance to penicillin and tetracycline, respectively [5]. β-lactamase (penicillinase) producing *N. gonorrhoeae* (PPNG) strains all express the TEM type β-lactamases encoded by closely related plasmids of slightly different types, named after geographical areas where they were first described (Asian; African; recently described Canadian subtype; Rio/Toronto; Nimes; Johannesburg; New Zealand and Australian) [28,29,30]. Although TEM-type β-lactamases expressed from these plasmids are not active against ESCs, the Asian-type TEM-135 variant is highly prone to evolving into an extended-spectrum β-lactamase, since it requires just a single amino acid substitution to develop the ability to hydrolyse ceftriaxone and cefixime [31]. Specifically altered *tetM* determinant, which mediates high tetracycline resistance in gonococci, blocks the tetracycline from attaching to its target. There are two different *tetM* determinants; American and Dutch, carried by the correspondingly named conjugative plasmids found in gonococci [29]. For more detailed information related to antibiotic resistance mechanisms in gonococci, the suggestion is a respected review by Unemo et al. [6].

## 3. WHO Surveillance

Considering the global problem of gonococcal AMR is worsening, the WHO is currently implementing a global intervention plan. It involves effective early prevention, diagnosis, sexual partner notification, raising awareness of correct antimicrobial use and treatment based on testing susceptibility, the establishment of effective drug regulations and prescription policies worldwide, research into novel treatment options and vaccine(s), and strengthened regular, quality-assured and comparable national and international AMR surveillance. The surveillance includes systematic monitoring of treatment failures and the use of molecular AMR prediction methods. The WHO recommends that gonorrhoea management guidelines should be revised based on recent and quality-assured gonococcal AMR surveillance data and that first-line empirical gonorrhoea treatment should be discontinued when the level of treatment failures and/or in vitro AMR reach 5% [32]. However, the evidence for the ≥ 5% AMR threshold is limited, and proportions of ≥ 1% and > 3% resistance in high-frequency transmitting populations has also been suggested [2]. The WHO Gonococcal Antimicrobial Surveillance Program (GASP) is of particular importance in dealing with gonococcal AMR. WHO GASP consists of networked reference laboratories interconnected with other international and national programmes, including the European Gonococcal Antimicrobial Surveillance Program (Euro-GASP), the US Gonococcal Isolate Surveillance Project (GISP), the Canadian GASP, the Gonococcal Antimicrobial Susceptibility Surveillance Program—Argentina (GASSP), the UK Gonococcal Resistance to Antimicrobials Surveillance Program (GRASP), and the Australian Gonococcal Surveillance Program (AGSP) [33,34,35,36,37]. The aims include collecting ≥100 representative gonococcal isolates per country and year, ideally using quantitative methods for the determination of the minimum inhibitory concentration (MIC) of antimicrobials using the agar dilution method or MIC gradient test strips and validated and standardized interpretative criteria. Most WHO GASP countries use the breakpoints of the European Committee on Antimicrobial Susceptibility Testing [38]. As far as molecular genotyping of *N. gonorrhoeae* AMR is concerned, a particularly important breakthrough was the relatively recent development of the respective sequence typing tool (https://ngstar.canada.ca, accessed on 5 December 2021), which assigns sequence types (STs) and alleles based on polymorphisms in *penA*, *mtrR*, *porB*, *ponA*, *gyrA*, *parC*, and 23SrRNA. These *N. gonorrhoeae* STs for antimicrobial resistance are now commonly used and combined with the STs assigned by the *N. gonorrhoeae* multiantigen sequence typing (http://ng-mast.net, accessed on 5 December 2021) and multilocus sequence typing (https://pubmlst.org/neisseria, accessed on 25 July 2022) methods [2].

Even after the expansion of WHO GASP, major concerns regarding the lack and quality of AMR data and its surveillance remained in many countries worldwide, most predominantly in the WHO African region (WHO AFR) and Eastern Mediterranean Region (EMR), but also in some countries of the former Soviet Union and South-Eastern parts of the WHO European region [39]. In the South-Eastern Asian (SEAR) and the Western Pacific Region (WPR), a substantial increase in the number of countries providing surveillance data for both ceftriaxone and azithromycin has been evident, as well as across the Asia–Pacific region. However, much more needs to be done, since many ceftriaxone-resistant strains originated from Asia–Pacific [2]. Concerns regarding the quality of data include low numbers of isolates (<100 isolates tested annually) and their selection, limited information about the total number of isolates and gonococcal diagnostic tests (culture and nucleic acid amplification tests—NAAT), as well as used typing methods. Many countries with limited gonococcal AMR surveillance have high incidences and suboptimal diagnosis of gonorrhoea, over-the-counter access to antimicrobials, and importantly limited availability of optimal AMR data supported by national antimicrobial treatment regimens. In several countries in these regions, WHO-GASP has overseen and financed efforts to strengthen and systemize the national gonococcal AMR surveillance programs, establish and/or broaden the capacities of referent microbiology laboratories and define AMR data-based nationwide treatments [2].

## 4. Current Surveillance Data in the WHO Regions

**Figure 1:**
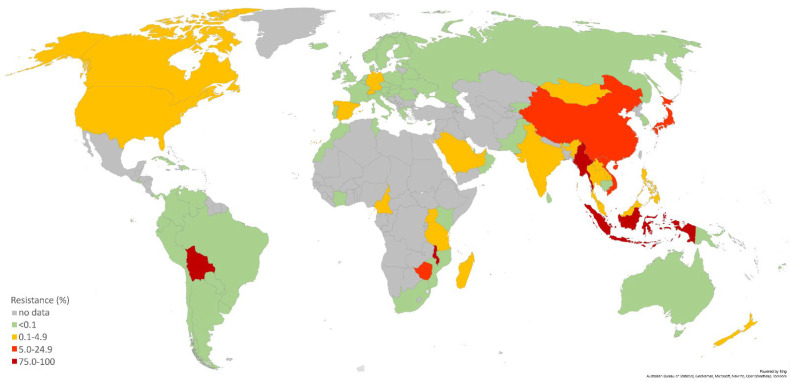
The percentage of isolates with decreased susceptibility or resistance to ceftriaxone according to the most recent data available for represented countries. Data were collected from references: [16,30,40,41,42,43,44,45,46,47,48,49,50].

**Figure 2:**
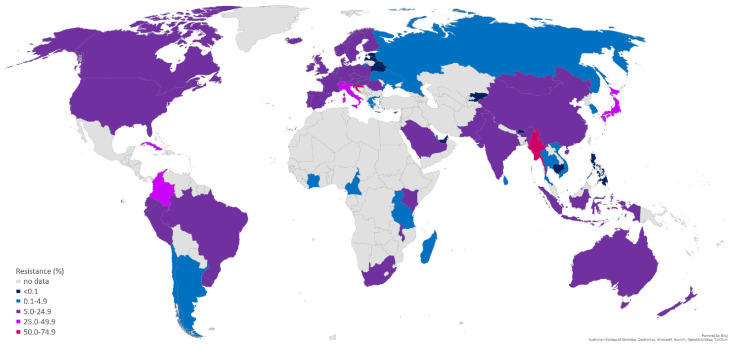
The percentage of isolates with decreased susceptibility or resistance to azithromycin according to the most recent data available for represented countries. Data were collected from references: [16,30,41,42,44,46,47,48,50,51].

The following literature overview aims at presenting the newest, mostly WHO-initiated, overseen and financed studies, which served to establish or reinforce the continual, quality-assured and comparable AMR surveillance, including systematic monitoring of treatment failures and use of molecular AMR prediction methods in WHO regions where major concerns regarding the lack and quality of AMR data and its surveillance are most pronounced—the WHO African region, the Eastern Mediterranean region, as well as the non-EU/EEA countries of the WHO European region (Figure 1 and Figure 2).

### 4.1. WHO European Region

Back in 2004, the European Centre for Disease Prevention and Control (ECDC) introduced the Euro-GASP, a system intended to provide studious and continual *N. gonorrhoeae* AMR monitoring. Euro-GASP now provides annual, quality-assured analyses of AMR data, connecting them with epidemiological and clinical data on gonorrhoea patients [40,52,53,54,55]. However, the last Euro-GASP study covered 27 of the EU/EEA countries, which still represents less than 50% of all the countries in the WHO European Region [15]. There is an exceeding scarcity of valid, quality assured and internationally reported AMR data in the non-EU/EEA countries. This represents a considerable problem since many of those countries have a relatively high burden of gonorrhoea, suboptimal diagnostics and surveillance of both the number of gonorrhoea cases and AMR [31,36]. Furthermore, the knowledge of gonococcal AMR remains limited even if awareness of it is well-existent, and antimicrobials of many different types, sources and quality are used for gonorrhoea treatment. This is particularly characteristic of the former Soviet countries, while in South-Eastern Europe situations vary, but in most countries, antibiotics cannot be procured without a prescription, and the treatment protocols are kept compliant with the newest WHO and EU recommendations [39]. The exception to the general situation in the former Soviet countries is the Russian Federation, which runs the quality-assured and WHO-standardized national Russian GASP (RU-GASP) programme of AMR surveillance. International comparability of AMR data from RU-GASP is assured, by the use of the 2008 WHO *N. gonorrhoeae* reference strains as quality controls as well as by the use of an external quality assessment (EQA) programme to ensure comparability of data [39].

The first two Euro-GASP surveys of gonococcal isolates from 2009–2010 and 2013, were focused on molecular epidemiological characterization by *N. gonorrhoeae* multi-antigen sequence typing (NG-MAST) in the earlier study, followed by whole-genome sequencing (WGS) of the isolates, in the latter. The distribution of particular sequence types and AMR clones across different patient groups was established [41,54]. The earlier study showed that the major EU/EEA lineage was *N. gonorrhoeae* 1407 (G1407), associated with decreased susceptibility and resistance to ESCs, mostly in men who have sex with men (MSM). This lineage threatened the recommended empirical cefixime monotherapy in the EU/EEA, so, in 2012, cefixime monotherapy was replaced by dual, ceftriaxone plus azithromycin therapy [56]. In 2013, the NG-MAST G1407 incidence had substantially decreased, and its association switched from MSM to heterosexual groups, particularly heterosexual women [55]. Since the 2012 recommendation was established, the incidence of resistance to cefixime and ceftriaxone has decreased, but the level of azithromycin resistance has increased [41,57]. This indicated that treatment recommendations, antimicrobial overuse and misuse, together with variations in patterns of sexual behaviours and networks, as well as expansions and eradication of specific gonococcal lineages, largely influenced the *N. gonorrhoeae* AMR situation in EU/EEA [58]. In the latest study, new WGS results on 2018 *N. gonorrhoeae* isolates were connected with quality-assured AMR data and epidemiological and clinical patient metadata [15]. Together with a longitudinal comparison with the two previous Euro-GASP surveys, this led to the observation of novel AMR lineages in the EU/EEA. Most importantly, the results of this study showed the emergence and subsequent expansion of an azithromycin-resistant clone (NG-MAST G12302), which explains the recent increase in azithromycin *N. gonorrhoeae* resistance. That clone carries the mosaic *mtrR* promoter and *mtrD* sequences and was associated with pharyngeal infections among MSM (NG-STAR CC63). Importantly, the decrease in cefixime resistance and increase in ceftriaxone susceptibility were also shown and linked with the progressive disappearance of a formerly predominant, extended-spectrum cephalosporin-resistant and mosaic *penA-*carrying NG-MAST G1407 (NG-STAR CC90) clone. It was suggested that the consequences of the emergence of an azithromycin-resistant NG-MAST G12302 clone may include a threat to the effectiveness of the currently recommended dual treatment for gonorrhoea. However, it was also highlighted that cases of ceftriaxone resistance in the EU and EEA are still rather scarce and that combined ceftriaxone and azithromycin resistance is exceedingly rare. Furthermore, most azithromycin-resistant isolates from this study had low-level azithromycin resistance [15].

In the EU/EEA, by far the best sustained time-wise is the ciprofloxacin resistance. Namely, the gonococcal population in the EU/EEA includes several lineages that have acquired and maintained several specific *gyrA* mutations which all cause severe ciprofloxacin resistance. Particularly worrisome is the presence of *parC* D86N mutation in some of these ciprofloxacin-resistant lineages. This mutation does not substantially increase the MIC of ciprofloxacin itself. Nevertheless, when S91F mutation in the main ciprofloxacin target, *gyrA* is present, this particular *parC* mutation increases the ciprofloxacin MICs and may predispose the development of resistance to any telomerase-inhibition-based new therapies, such as gepotidacin. Gepotidacin is targeting GyrA and ParC and gonococcal isolates with the *parC* D86N mutation can select for gepotidacin-resistance mutation in the GyrA target (*gyrA* A92T) during treatment, thereby potentially causing clinical gepotidacin resistance [16,17]. Fortunately, no such mutations were found in the Euro-GASP 2018 dataset, meaning that the gonococcal population of EU/EEA appears to be susceptible to gepotidacin. Zoliflodacin resistance mutations in *gyrB* D429 and K450 have been selected in vitro. However, in the Euro-GASP 2018 material, no such mutations were found, indicating full susceptibility to zoliflodacin [59]. It was recently shown that *N. gonorrhoea* zoliflodacin resistance-conferring region located in *gyrB* can be taken up from commensal *Neisseria* species by horizontal gene transfer, most significantly via transformation. This constitutes a rationale for conducting surveillance of zoliflodacin susceptibility in commensal *Neisseria* as well, to prevent the emergence and rise of zoliflodacin AMR in gonococci, since it was indicated that its over-use may increase the corresponding AMR genes reservoir [60].

Interestingly, in the Russian Federation, the use of benzylpenicillin, along with penicillins of subsequent generations for gonorrhoea treatment was abandoned relatively recently [61]. Namely, in 2010, the RU-GASP reported a high prevalence of resistance to penicillin (32%), tetracyclines (42%) and ciprofloxacin (53%), and a relatively high prevalence of resistance to azithromycin (4.9%) and spectinomycin (4.4%) [39]. Accordingly, penicillin is now no longer used for gonorrhoea treatment in Russia, and, at present, the antibiotics recommended are ceftriaxone and spectinomycin. Unlike the EU/EEA countries, azithromycin has never been recommended for the treatment of gonorrhoea, and cefixime has not yet been introduced into medical practice [61]. The most recent results of *N. gonorrhoeae* surveillance in Russia within the framework of the RU-GASP Programme indicated decreasing trends in the resistance to the antibiotics previously used for gonorrhoea treatment (benzylpenicillin, tetracycline, and ciprofloxacin) [62,63,64]. Partial recovery of *N. gonorrhoeae* tetracycline susceptibility was observed, relative to the proportion of isolates with resistance detected ten years prior. However, the levels of tetracycline resistance still remained high enough to preclude their use for gonorrhoea therapy [62]. Meanwhile, isolates with slightly decreased susceptibility to ceftriaxone appeared only sporadically. The latest data on susceptibility and genetic determinants of resistance to benzylpenicillin and ceftriaxone in *N. gonorrhoeae* in Russia during the 2015–2017 period revealed that 7.7% of isolates were resistant to benzylpenicillin, and 47.5% showed intermediate resistance. The accumulation of mutations in chromosomal genes (*penA*, *pon*, *porA*, and *mtrR*) led to a stepwise increase in penicillin resistance up to the intermediate level. Only one ceftriaxone-resistant isolate and two isolates at the resistance breakpoint were found. This suggested that the recommendation to use it as a first-line drug for gonorrhoea monotherapy in the Russian Federation should be maintained. Additional analysis of samples with maximum MICcef values, including sequencing of *penA*, did not reveal the mutations associated with resistance to ESCs and showed a non-mosaic structure of *penA* [61]. One of the interesting facts observed was the simultaneous presence of the β-lactamase-producing (*bla*TEM) and *tetM* plasmids associated with high resistance to penicillins and tetracyclines. Previous studies have shown that the conjugative *tetM* plasmid in *N. gonorrhoeae* facilitates the acquisition of other plasmids by the cell [65,66]. Thus, the analysis of drug resistance determinants calls for special attention to tetracyclines resistant isolates and carrying *tetM* plasmids, because the presence of this genetic element simplifies the transfer of *bla*TEM plasmids and other plasmids containing genes associated with resistance to other antimicrobial drugs.

The data from Ukraine’s Ternopil and Dnipropetrovsk regions from 2013–2018 displayed that 9.3% of isolates were resistant to ciprofloxacin, 6.0% to tetracyclines, 2.0% to azithromycin, and 0.7% to benzylpenicillin. No isolates were resistant to ceftriaxone, cefixime, spectinomycin, or gentamicin. However, one isolate showed a bordering resistance for both ceftriaxone and cefixime. The conclusion was that ceftriaxone should remain an empiric first-line treatment in Ukraine, in dual therapy with azithromycin or doxycycline or in monotherapy. An important limitation was the fact that only two regions of Ukraine were monitored, in addition to the lack of data on the corresponding molecular and genotypic determinants of AMR. Importantly, one of the major problems in AMR control in Ukraine is the easy, no-prescription-needed access to antibiotics, all over this country [40].

The most recent study from Belarus for the period 2009–2019 regarded AMR reports that overall, 52.2% of patients received nation-wide recommended first-line treatment (ceftriaxone 1 g), 9.3% received the alternative (cefixime 400 mg or ofloxacin 400 mg), and 38.5% were given the non-recommended treatment [67]. Interestingly, very different results from the ones described in Ukraine were acquired. Namely, 27.8% of the isolates were resistant to tetracycline, 24.7% to ciprofloxacin, 7.0% to benzylpenicillin, 2.7% to cefixime, and 0.8% to azithromycin. No isolates were resistant to ceftriaxone, spectinomycin, or gentamicin. However, 2.7% of isolates had a ceftriaxone MIC exactly at the resistance breakpoint. Only one isolate produced β-lactamase. During the study, the levels of resistance to ciprofloxacin and tetracycline remained relatively high and stable. Resistance to cefixime was unidentified before 2013 but peaked at 22.2% in 2017. Only two isolates with resistance to azithromycin were found recently (2018–2019). No resistance to ceftriaxone was identified. The conclusion was that ceftriaxone 1 g should be the recommended first-line gonorrhoea therapy in Belarus, while the use of fluoroquinolones should be limited only to patients with confirmed susceptibility. Evidence-based gonorrhoea treatment guidelines and continued surveillance of gonococcal AMR in Belarus were stated as imperative [67].

### 4.2. WHO Western Pacific Region (WPR)

The latest data from this region report the prevalence and molecular mechanisms of ESC resistance of *N. gonorrhoeae* isolates in 2017 in Shanghai [68]. Approximately 20% of the isolates exhibited cefixime resistance, and 5.5% were ceftriaxone resistant. The most frequent resistance determinant (72.7% of ESC resistant isolates) was clonally distributed and associated with mosaic *penA* (10 or 60) alleles, while non-mosaic *penA* 18 allele, substitutions in *penA* A501T, G542S, as well as *porB* G213S/Y mutation were nonclonal. Furthermore, 6.8% of the isolates showed decreased susceptibility to azithromycin and were associated with 23S rRNA A2059G mutation. Almost all isolates were ciprofloxacin resistant and linked with *gyrA* 91/92 and *parC* 85/86/87/88/89/91 alterations. Isolates with *parC* S88P substitution were clustered into the ESC-resistant clade. 

Particularly informative from the point of view of sensitivity monitoring and comparisons of a novel with the effectivity of older therapeutics was the study performed between 2014 and 2018 in Nanjing [42]. All isolates were susceptible to spectinomycin and resistant to ciprofloxacin, 21.2% were resistant to azithromycin, 43.4% to penicillin, 26.9% were tetracycline-resistant, while 19.4% were multidrug-resistant isolates. The MICs of zoliflodacin ranged from 0.002 to 0.25 mg/L, with a twofold increase in MICs in 2018 compared with 2014. Isolates with the lowest (0.002–0.015 mg/L) and highest (0.125–0.25 mg/L) zoliflodacin MICs were also quinolone-resistant with double or triple mutations in *gyrA*, and 95.5% of them also had mutations in *parC*. No D429N/A and/or K450T mutations in *gyrB* were identified in isolates with higher zoliflodacin MICs, S467N mutation in *gyrB* was identified in one such isolate. Despite the yearly changes indicating a constant decline of susceptibility to zoliflodacin, its excellent in vitro activity manifests continuously against clinical gonococcal isolates, including those with high-level resistance to ciprofloxacin, azithromycin, and extended-spectrum cephalosporins.

### 4.3. WHO African Region

The region has had minimal or rudimentary gonococcal monitoring systems with data on susceptibility, genotypes and phenotypes of resistance remaining largely unknown. Recently, the WHO has attempted to broaden the systemic surveillance of gonorrhoea AMR by initiating Enhanced Gonococcal Antimicrobial Surveillance Program (EGASP) but the number of countries able to implement it remained relatively small [43]. However, several good examples have emerged lately in Sub-Saharan Africa. One of them is Uganda, where EGASP was initiated to monitor *N. gonorrheae* resistance trends. In line with this, a study of a large surveillance program of men with urethral discharge syndrome from Kampala was performed, and a practically first report from Uganda was published [44]. Data for the period 2016–2018 revealed that the resistance rate to ciprofloxacin, tetracycline, and penicillin was extremely high, over 90% and that 96% of isolates were azithromycin-susceptible. Results demonstrated the resistance or decreased susceptibility to all key anti-gonococcal antibiotics except azithromycin. The conclusion was that there is poor antibiotic stewardship, near-universal resistance to many antimicrobials, with emerging resistance to others, which all suggested a need for permanent surveillance efforts to curtail antimicrobial-resistant gonorrhoea in Uganda.

Another example of good AMR surveillance is that in South Africa, conducted in all of its nine provinces. The report from Johannesburg categorised one-third of the isolates as multidrug-resistant. Importantly, 15% of the isolates showed resistance to azithromycin and all had mutations in the *mtrR* promoter [45]. The reported rates of azithromycin resistance are not consistent across the entire country [45,46]. Research performed on both men and women from the province of KwaZulu-Natal discovered azithromycin resistance in 68% of tested isolates, with 71% being multidrug-resistant [46]. These disparities indicate a necessity for a concerted effort to establish the real extent of azithromycin resistance across South Africa, especially since current syndromic management with dual ceftriaxone and azithromycin therapy would factually be rendered a monotherapy if the rate of azithromycin resistance was that high [46].

Initiative for robust AMR monitoring exists in Cameroon as well, particularly after the establishment of Centre Pasteur du Cameroun’s laboratory information system. Data for the period 2012–2018 report resistance to ciprofloxacin (64.4%), benzylpenicillin (80.1%), and tetracycline (58.4%). Most importantly, ciprofloxacin resistance grew dramatically—from 15.0% in 2012 to 79.5% in 2018. Resistance to benzylpenicillin has decreased significantly since 2016 and the resistance to tetracycline has remained steady. The prevalence of resistance to ceftriaxone, azithromycin, and spectinomycin was low. It was confirmed that ciprofloxacin should not be recommended anymore as a first-line treatment for gonorrhoea in Cameroon and advocated for the establishment of a sustainable gonococcal antimicrobial surveillance programme to control gonorrhoea [47].

In Kenya, data on gonococcal antimicrobial resistance are limited. In a study conducted between 2013 and 2018, 66.7% of isolates had both β-lactamase (TEM) and *tetM* encoding plasmids. Moreover, 86.1% of isolates contained *tetM* encoding plasmids with MIC > 1 mg/L, and all analyzed isolates had non-mosaic *penA* genes. Furthermore, all isolates had S10 ribosomal protein V57M amino acid substitution associated with tetracycline resistance. Thus, high-level gonococcal penicillin and tetracycline resistance is mediated by plasmid-borne *bla*TEM and *tetM* genes. While the African TEM plasmid, TEM1 and American *tetM* are the dominant genotypes, Asian TEM plasmid, new TEM239 and Dutch *tetM* have just emerged. The ESC resistance-conferring *penA* alleles were not observed. AMR determinants of macrolide resistance were absent, as only 8.3% of tested expressed a low-level azithromycin resistance. The use of penicillin and tetracycline for gonorrhoea treatment was stopped many years ago in Kenya, and the absence of molecular markers associated with high levels of azithromycin, cefixime and ceftriaxone resistance, shows that these antibiotics could still be useful for the treatment of gonococcal infections in Kenya [29].

### 4.4. WHO Eastern Mediterranean Region (EMR)

Similarly to WHO Africa, limited data on AMR in *N. gonorrheae* strains circulating in the EMR are available. The treatment of gonococcal infections in Morroco was recently changed by replacing ciprofloxacin with single-dose (250 mg) ceftriaxone, and a nationwide gonorrhoea surveillance system was introduced. This change was based on a study in men with gonorrhoea, in which high rates of penicillin, tetracycline and ciprofloxacin resistance were found (56.2%, 92.6%, and 86.8%, respectively), together with a lack of resistance to spectinomycin, ceftriaxone or cefixime [48]. The molecular mechanisms of AMR were conducted among HIV-negative women from Fez between 2013–2015, most of whom (68.5%) had genitally asymptomatic gonorrhoea [51]. An interesting finding was the high frequency (36.1%) of samples with the simultaneous presence of both Toronto and Asian types of β-lactamase-encoding plasmids, which was indicative of the high rate of mixed gonococcal infections. The *tetM* plasmids were present in 49.5% of cases, most of which were American type 93% and only 7.3% were Dutch. Furthermore, the majority of the β-lactamase-producing positive samples (61.1%) also carried the *tetM*-encoding plasmid, which is in agreement with the old hypothesis that the *tetM* plasmid plays a role in acquiring the β-lactamase-producing plasmids [69]. Finally, 77.9% of isolates showed the mutation Ser-91 in *gyrA*, extrapolating to ciprofloxacin resistance. Apart from showing that plasmid genotyping can be used in the detection of mixed gonococcal infections during AMR surveillance, the presence of mixed infections is indicative of the sexual behaviour patterns of patients and/or their partners, being that individuals with multiple sexual partners are at a higher risk of mixed NG infections. This shows the current failure of management of *N. gonorrhoea* and other STIs in urban areas of Morocco, but ultimately confirms that the chosen regime of gonorrhoea treatment with ceftriaxone monotherapy is the right choice, at present [51].

In the Arabian Peninsula, the most recent first-time characterization of antimicrobial susceptibility and resistance of *N. gonorrheae* isolates was done in Qatar between 2017–2020 [49]. Resistance in isolates from urogenital sites of patients was 64.7% for ciprofloxacin, 50.7% for tetracycline, and 30.8% for benzylpenicillin. The percentage of isolates non-susceptible to azithromycin was 4.1% and all isolates were susceptible to ceftriaxone. High-level resistance to azithromycin had 1.6% isolates from 2019 and one isolate from 2020. Overall, 1% of isolates had ceftriaxone MICs at the susceptibility breakpoint (0.25 mg/L). A recommended gonorrhoea therapy was continual dual treatment with ceftriaxone (250 mg) plus azithromycin (1 g).

## 5. New Treatment Options for *N. gonorrhoea* Infections

Since the systemic establishment of effective drug regulations and prescription policies, together with serious monitoring of treatment failure, still seem far off in most settings, raising cognizance of the correct antimicrobial use combined with the strictly controlled antibiotic prescription policies must also be applied globally if any kind of success of the WHO plan of determent of the gonococcal AMR to widely available antimicrobials is to be expected. Even if all that is accomplished, the impressive repertoire of resistance determinants *N. gonorrhoeae* has developed owing to its high ability to obtain them, creates a constant need for a proactive search for new treatment avenues.

The most promising new antibiotics gepotidacin and zoliflodacin were shown to mostly be effective against the non-complicated infections seen in urogenital gonorrhoea, but not for infections of the pharynx [5]. Furthermore, indications of gepotidacin cross-resistance with ciprofloxacin are also worrying, particularly in populations with higher incidences of ciprofloxacin resistance, such as the WPR. Namely, all gepotidacin-resistant isolates contain ciprofloxacin resistance-conferring *parC* D86N and *gyrA* S91F-D95A/G polymorphisms, and *parC* D86N predisposes them to also gain specifically gepotidacin resistance-conferring *gyrA* or A92T polymorphism [5,16,17]. In the latest EU/EEA-based clinical study, such mutations were not found, indicating the susceptibility of the gonococcal population of EU/EEA to gepotidacin [15]. Zoliflodacin resistance mutations in *gyrB* (D429 and K450) were absent as well, indicating full susceptibility to zoliflodacin in EU/EEA [59]. If fully approved after current phase III trials, these new antimicrobials may show as important treatment avenues for urogenital infections in the mostly high-income and developed countries of the EU/EEA. However, sexual networks and behaviours in this and most other developed regions favour the rise in the incidence of complications and pharyngeal infections. Hence, continued research on repurposing already clinically approved antimicrobials remains imperative. Extensive studies and clinical trials were only done for gentamicin, but its efficacy has proven to be sub-optimal, particularly for pharyngeal infections. Importantly, in a recent study of the relative efficacy of high-dose intravenous ceftriaxone and oral cefixime combined with doxycycline for treatment of the frequently seen and sexually transmittable *Chlamydia trachomatis* and *Neisseria gonorrhoeae* co-infection, the combination of doxycycline with ceftriaxone was indicated as superior to its combination with cefixime, and cefixime with doxycycline was recommended only in patients with ceftriaxone allergy and in settings where ceftriaxone is unavailable [50]. Of the already approved therapeutics, the most promising seems to be ertapenem. Ertapenem has shown particularly encouraging activity, most prominently as a potential substitution for ceftriaxone and cefixime [4,70]. Namely, it was shown that ceftriaxone-resistant isolates, particularly, highly resistant *penA* 37 and 42-containing HO41 and F89 strains were susceptible to ertapenem. Most importantly, the *penA* 60 containing and internationally spreading FC428 clone appeared to be susceptible to ertapenem, as well. Ertapenem has also been successfully used for the treatment of strains displaying combined ceftriaxone and high-level azithromycin resistance. Of note, poor cross-resistance and interactions between ceftriaxone and ertapenem were shown, indicating that ertapenem may not just be a good substitution, but also a good supplement in combinatorial therapies with ceftriaxone, thus possibly replacing azithromycin in regions with the corresponding high-level or high-incidence resistance [5]. In the most recent study of ertapenem activity against isolates with decreased susceptibility to ESCs, however, a trend of linear increase of isolates with ertapenem MICs higher or equal to those of ceftriaxone was seen, with the characteristic mosaic *penA* allele present in a significantly higher proportion of such isolates. Nevertheless, it was indicated that ertapenem should be effective in ESC-resistant isolates [70].

Notably, all clinical trials for ESC-alternative gonorrhoea therapies over the past decade have been performed in the USA, UK, and Australia, while antimicrobial resistance and the presence of resistance determinants are much more problematic in the WPR. The latest study of zoliflodacin resistance from clinical isolates collected in Nanjing, China indicates excellent in vitro activity including the ones with high-level resistance to ciprofloxacin, azithromycin, and ESCs. An important and worrying finding was that susceptibility to zoliflodacin annually declined during the study period. This indicates that, as the usage of novel treatments such as zoliflodacin progress, they form a more significant selective pressure. The incidence of the already existent and newly arising resistance-conferring polymorphisms will inevitably arise and the decrease in susceptibility of *N. gonorrheae* to these treatments will emerge worldwide [42].

Overall, it seems unlikely that alternative first-line ceftriaxone replacements for gonorrhoea treatment will become available soon. For now, elevated ceftriaxone doses of 1–2 g seem to be sufficient for gonorrhoea treatment, since even in China they have largely preserved treatment success [5]. However, results from Shanghai show a high overall percentage (more than 72% of isolates) of resistance to the most important ESCs. Of these, 5.5% were ceftriaxone resistant and associated with mosaic *penA* genes [68]. Although ertapenem seems like the best option to replace ceftriaxone, considering the susceptibility of high-level resistant strains to this β-lactam, many more extensive studies need to be conducted for this to be considered. A most likely future scenario is the individualized treatment of gonorrhoea, based on specific antimicrobial susceptibility levels determined for each particular isolate. Rapid molecular tests detecting *N. gonorrhoeae* together with AMR determinants for multiple drugs should become available. These tests could be used for prompt diagnosis, and AMR surveillance, as well as to guide individualised treatments at the first healthcare visit [2]. The AMR surveillance would be integrated into routine diagnostics and used to rectify the treatment recommendations, by directing appropriate treatments and evading last-line antimicrobials, hence minimizing the emergence and spread of AMR. Naturally, continued research to describe novel AMR determinants, and their emergence, evolution and biological fitness are needed [2].

## 6. Conclusions

Although progress is achieved owing to WHO GASP, gonococcal AMR remains a major global public health concern, compromising treatment and control of gonorrhoea. National and international leadership and financial commitment are needed for WHO GASP to be sustained, further expanded and improved, in order to enable the AMR data-based monitoring of gonorrhoea, combined with the development of prescription policies toward correct and conscious antimicrobial use. Research enabling novel antimicrobials to be fully approved and/or conserved for gonorrhoea treatment by AMR surveillance, as well as an exploration into and forming of new diagnostic tests for simultaneous diagnosis and surveillance of gonococcal AMR is also needed. Until vaccines are completely developed, prevention should be aimed at education regarding symptomatic and asymptomatic STIs, promotion of safe sexual behaviours, enhanced sexual partner notification and treatment, and expansion of targeted interventions. This should ideally be linked to the general prevention of STIs, as well as combined with testing oriented towards the detection of asymptomatic urogenital gonorrhoea in women and pharyngeal gonorrhoea in both sexes, and, finally, integrated with the management of gonorrhoea by continual informed changes in public health policies and treatment guidelines.

## Data Availability

Not applicable.
